# Nutrient disturbance in a shallow aquaculture pond impacts *Microcystis* gene expression but does not impact bacterial community function during bloom conditions

**DOI:** 10.1111/jpy.70197

**Published:** 2026-07-09

**Authors:** Matthew F. Gladfelter, Hunter R. Baylous, Alan E. Wilson, Morgan M. Steffen

**Affiliations:** ^1^ School of Fisheries, Aquaculture, and Aquatic Sciences Auburn University Auburn Alabama USA; ^2^ Department of Biology James Madison University Harrisonburg Virginia USA

**Keywords:** cyanobacteria, eutrophication, freshwater, phycosphere, transcriptomics

## Abstract

Cyanobacterial harmful algal blooms (cHABs) are worldwide issues. Reduced nitrogen forms (ammonium and urea) have recently been measured in freshwater systems at concentrations not previously recorded. These reduced nitrogen forms have been shown to favor the proliferation of harmful cyanobacteria. These blooms are comprised of a diverse community of microbes that contribute to nutrient cycling and other ecosystem functions. To measure the response of the *Microcystis* bloom microbiome, a field experiment was conducted to examine the transcriptional responses of *Microcystis*, a common bloom‐forming cyanobacterium, as well as the co‐occurring bacteria associated with the bloom. Limnocorrals were fertilized with either nitrate, ammonium, or urea, and samples were collected across a 24‐h time series after nutrient additions to track changes in *Microcystis* gene expression along with bacterial function and composition using metatranscriptomics. *Microcystis* spp. dominated experimental enclosures throughout the experiment (>70% of total bacterial reads). This stability was also reflected in the community structure and function of the co‐occurring bacteria, which had no substantial changes over the 24‐h after nutrient additions. Nutrient additions drove immediate differential expression responses for *Microcystis*, and the response was nitrogen form dependent. Key gene groups including core metabolite‐related genes and carbon acquisition genes had pronounced differences among treatments, while toxin‐related gene expression (*mcy*ABCDEFGHIJ) was not impacted by nitrogen treatments. Results support previous lab and field‐based experiments that have suggested that reduced nitrogen forms impact cHAB molecular physiology, even during peak bloom and elevated nutrient conditions in shallow systems frequently impacted by agricultural runoff and/or aquacultural input.

**Abbreviations**
ABC: adenosine triphosphate binding cassette
AMP: adenosine monophosphate
cHABs: cyanobacterial harmful algal blooms
dGMP: deoxyguanosine monophosphate
FDR: false discovery rate
GS/GOGAT: glutamine synthetase‐glutamine synthase
RM: restriction modification
RM‐ANOVA: repeated‐measures analysis of variance
ROS: reactive oxygen species
rRNA: ribosomal ribonucleic acid
SAICAR: phosphoribosylaminoimidazolesuccinocarboxamide
TN: total nitrogen
TP: total phosphorus

## INTRODUCTION

Cyanobacterial harmful algal blooms (cHABs) are global issues that are further intensified by inputs of excessive nutrients (nitrogen and phosphorus) from terrestrial systems (Anderson et al., 2002; Glibert et al., 2014; Paerl & Huisman, 2008). These blooms can drastically disrupt aquatic systems and be hazardous to wildlife, livestock, pets, and humans. Cyanobacterial scums can shade‐out competing primary producers and create hypoxic zones that suffocate aquatic species (Hallegraeff, 2003; Paerl et al., 2001). Bloom‐forming species may also produce taste and odor compounds that devalue the water and any fish within it and may release toxic secondary metabolites that can be serious health risks both acutely and chronically (Carmichael & Boyer, 2016; Paerl et al., 2001; Watson et al., 2016). *Microcystis* is a common bloom‐forming cyanobacterium that has been documented globally (Harke et al., 2016). Like many other cyanobacteria, *Microcystis* spp. have a suite of attributes that allow them to dominate freshwater ecosystems, including the ability to regulate buoyancy, a thick mucilage that buffers the colonies from parasites and grazers, a wide range of survivable temperatures, and the ability to store both nitrogen and phosphorus for future use, among others (Huisman et al., 2018; Paerl et al., 2001). Molecular‐based approaches have also identified another characteristic of *Microcystis* that further helps these organisms proliferate throughout freshwater systems: the co‐occurring bacteria that reside within the colony mucilage (Amin et al., 2015; Baker et al., 2022; Hoke et al., 2021; Seymour et al., 2017; Variem & Kizhakkedath, 2021). This microenvironment, often referred to as the phycosphere, is the area immediately surrounding algal cells or colonies that is typically high in nutrients and other organic molecules that are exchanged between the host phytoplankton and its associated microbiome (Seymour et al., 2017).

Co‐occurring bacteria in the phycosphere have been proven to break down harmful molecules and recycle nutrients (Amin et al., 2015; Fouilland et al., 2014; Hoke et al., 2021; Seymour et al., 2017). Indeed, the co‐occurring bacteria are capable of detoxifying reactive oxygen species (ROS) such as hydrogen peroxide, which can be lethal to the cyanobacterial cells (Morris et al., 2011; Seymour et al., 2017). Furthermore, depending on the freshwater system and species present, remineralized phosphorus and nitrogen can constitute a substantial amount of bioavailable nutrients that phytoplankton can utilize for growth and proliferation (Jin et al., 2023; Li et al., 2017; Shinohara et al., 2012). This production and recycling of vital macro‐ and micronutrients can be enough to sustain phytoplankton growth when allochthonous nutrient inputs are low (Cole, 1982; Seymour et al., 2017). However, these complex communities are still reliant upon incoming macronutrients for continued growth, and the form and concentration of these macronutrients have been shown to change phycosphere interactions in laboratory experiments (Ma et al., 2018; Mandal et al., 2018; Perera et al., 2022).

Previous studies have shown how *Microcystis* and other phytoplankton taxa respond biologically (An et al., 2020; Deschoenmaeker et al., 2017; Donald et al., 2011; Erratt et al., 2018, 2020; Gladfelter et al., 2022; Khazi et al., 2018; Rückert & Giani, 2004) and transcriptionally (Harke & Gobler, 2013, Harke & Davis, et al., 2015; Steffen et al., 2014) when given different forms of macronutrients (nitrogen and phosphorus) in variable concentrations. Although nitrogen and phosphorus are both crucial for proper cell growth and function, nitrogen can be available to phytoplankton in many different forms from allochthonous runoff, namely, nitrate, ammonium, or urea. Nitrate has historically been the most common nitrogen form in anthropogenic runoff due to its popularity as an agricultural fertilizer. However, in recent decades, urea has been by far the most widely used agricultural fertilizer worldwide (Glibert et al., 2006, 2014). This increased use has resulted in reduced nitrogen forms (ammonium and urea) being observed in freshwater systems at concentrations not previously recorded. This is particularly troubling considering the growing body of evidence that suggests reduced nitrogen forms encourage greater cyanobacterial biomass and are stored more efficiently, allowing the bloom to sustain itself for longer compared to oxidized nitrogen forms (i.e., nitrate and nitrite; Deschoenmaeker et al., 2017; Gladfelter et al., 2022; Glibert et al., 2006, 2014; Khazi et al., 2018).

Cyanobacteria have very different uptake and assimilation pathways with regard to these different forms of nitrogen (i.e., nitrate, ammonium, and urea; Herrero et al., 2001). It is generally accepted that each of these nitrogen forms has its own energetic cost associated with incorporating that nitrogen source into the cyanobacterial cell (Donald et al., 2011; Erratt et al., 2018; Herrero et al., 2001). Oxidized forms of nitrogen (i.e., nitrate and nitrite) each require biochemical reduction to ammonium, which is costly compared to reduced forms of nitrogen (i.e., ammonium and urea). These reduced nitrogen forms can either passively diffuse into the cell and be ready to use (ammonium) or require minimal alteration but result in two ammonium molecules for every one molecule of nitrogen source (urea; Donald et al., 2011; Herrero et al., 2001). Therefore, examining how the entire bloom community, including the co‐occurring bacteria, can help shed light on the advantages that cyanobacteria gain when using reduced nitrogen for growth.

Here, we conducted a field experiment within a small and shallow (13.233 m^2^ surface area and 1.83 m deep) aquaculture pond that would allow us to better determine how a naturally formed *Microcystis* bloom community, including the co‐occurring bacteria, responds to nutrient additions in a system with greater exchange (nutrient and microbial) throughout the water column compared to larger lake systems (e.g., Lake Erie and Lake Taihu), thus advancing the understanding of *Microcystis* biology and molecular ecology simultaneously. This field experiment aimed to (1) examine the molecular response of *Microcystis* when supplemented with three common nitrogen forms—nitrate, ammonium, and urea—during peak bloom conditions, specifically, the changes in gene expression (up‐ and downregulation of key genes) and (2) assess how the co‐occurring bacteria within the phycosphere would respond to a sudden pulse of different nitrogen forms, representing potential episodic events such as storm runoff.

## MATERIALS AND METHODS

### Site description

This field experiment was carried out in late September of 2020 in pond S10, an earthen aquaculture pond located at Auburn University's E.W. Shell Fisheries Center in Auburn, Alabama, United States (32°40′8.929″ N, 85°30′31.464″ W). Pond S10 is an aquaculture production pond with an average depth of 1.83 m, a surface area of 13.233 m^2^, and an approximate volume of 22.819 m^3^ (Boyd & Shelton, 1984). The pond is categorized as hypereutrophic, with average annual total nitrogen (TN), total phosphorus (TP), and chlorophyll *a* values of 2587, 372, and 146 μg · L^−1^, respectively (A. E. Wilson, unpublished data). Initial TN, TP, and chlorophyll *a* values at the start of the experiment were 2000, 220, and 85 μg · L^−1^, respectively.

### Study design and sample collection

The experiment included twenty‐four 1100‐L plastic limnocorrals suspended in PVC frames and filled with water from pond S10. Limnocorrals were randomly assigned to one of three treatments, including nitrate addition, ammonium addition, and urea addition, plus control enclosures (*n* = 6 limnocorrals per treatment). All nitrogen was added to increase total nitrogen by 600 μg · L^−1^, and phosphorus was added to increase total phosphorus by 100 μg · L^−1^ to combat phosphorus limitation. For more details about experimental design, see Gladfelter et al. (2022).

Samples for molecular processing were collected immediately prior to treatment applications (0 h), 2 h post‐treatment (2 h), and 24 h post‐treatment (24 h). Water was collected from three mesocosms randomly selected from each treatment prior to the start of the experiment (*n* = 3 per treatment, *n* = 12 per sampling event, *N* = 36 libraries across the entire experiment). These three chosen enclosures were sampled throughout the experiment (i.e., the same 12 mesocosms were sampled during each sampling event). Biomass for mRNA was collected in situ by pushing whole water through a Sterivex‐GP pressure filter unit (MilliporeSigma, Burlington, Massachusetts, United States) using a 50‐mL syringe until no more water could pass through. The outlet of the Sterivex filter unit was plugged with clay, and the filter unit was filled with RNA*later* stabilization solution (ThermoFisher, Waltham, Massachusetts, United States) and placed in the dark, under dry ice until taken back to the lab, where filters were stored at −80°C before extraction and sequencing.

### Sample extraction and sequencing

Frozen samples were extracted using the Qiagen RNEasy PowerWater kit following the manufacturer's instructions. Filters were removed from the casing according to Cruaud et al. (2017). Contaminating DNA was removed, and samples were tested for purity as previously described (Baylous et al., 2024; Steffen et al., 2015). Quality, purity, library preparation, and sequencing were all performed by Azenta Genewiz® (South Plainfield, New Jersey, United States). rRNA reduction was performed using the Qiagen FastSelect rRNA 5S/16S/23S kit. Library preparation was performed using the NEBNext Ultra II RNA Library Preparation Kit for Illumina according to the manufacturer's instructions. Sequencing was performed on the Illumina HiSeq (4000 or equivalent) using the 2 × 150 bp paired end configuration. Samples were demultiplexed using Illumina bcl2fastq 2.17 and delivered as fastq files.

### Bioinformatic analysis

Reads were imported into CLC Genomics Workbench (Qiagen) as previously described (Steffen et al., 2014, 2015, 2017). Briefly, reads were paired and aligned to the *Microcystis aeruginosa* NIES 843 genome (Kaneko et al., 2007), and expression values were calculated as total per million (TPM) with the minimum length fraction (0.9) and similarity fraction (0.9) increased from default parameters. Coverage was calculated based on reads mapped to the NIES 843 genome during expression analysis. Differential expression was calculated using the differential expression for RNA‐seq tool in the RNA‐seq module. Baggerly's *t*‐test using a false discovery rate (FDR) corrected *p*‐value cutoff of 0.05 and fold change cutoff of ±1.75. For microbial community expression analysis, fastq files were imported into the MG‐RAST online analysis pipeline. Abundance hits for the phylum, class, and order data files from MG‐RAST using Refseq annotation were transformed into percent abundance for analysis. Functional characterization was performed in MG‐RAST using the subsystems (SEED) annotation analysis function using default settings. Heat maps for key functional groups were made using Primer 7 v. 7.0.24. Gene expression values (TPM) were log transformed (log[x + 1]) prior to generating figures to avoid null values. Bray–Curtis similarity analysis was used to cluster individual sample replicates and generate dendrograms.

## RESULTS

### Sequencing output

A total of ~1.52 billion reads were generated from 36 individual sequence libraries, with an average of 42.1 million reads per sample. An average of 46.6% of reads in each library mapped to the genome of *Microcystis aeruginosa* NIES 843. Average coverage based on total bp mapped to the *M. aeruginosa* NIES 843 genome was 591x (Table S1). According to libraries analyzed in MG‐RAST, there was an average of 1,033,855 hits per sample with 26.3% of those hits being attributed to *M. aeruginosa*, on average (Table S2).

### Community structure and function according to metatranscriptomes

**FIGURE 1:**
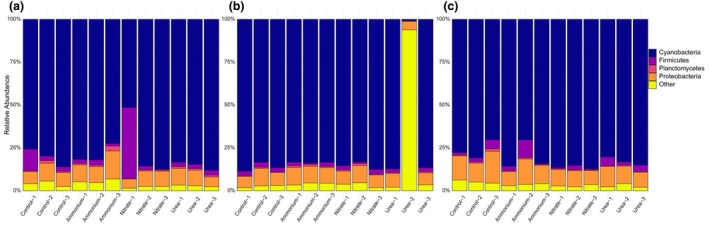
Relative abundance of major bacterial taxa before nutrient additions (a), 2 h after additions (b), and 24 h after additions (c). Relative abundance calculated based on sequence libraries.

**FIGURE 2:**
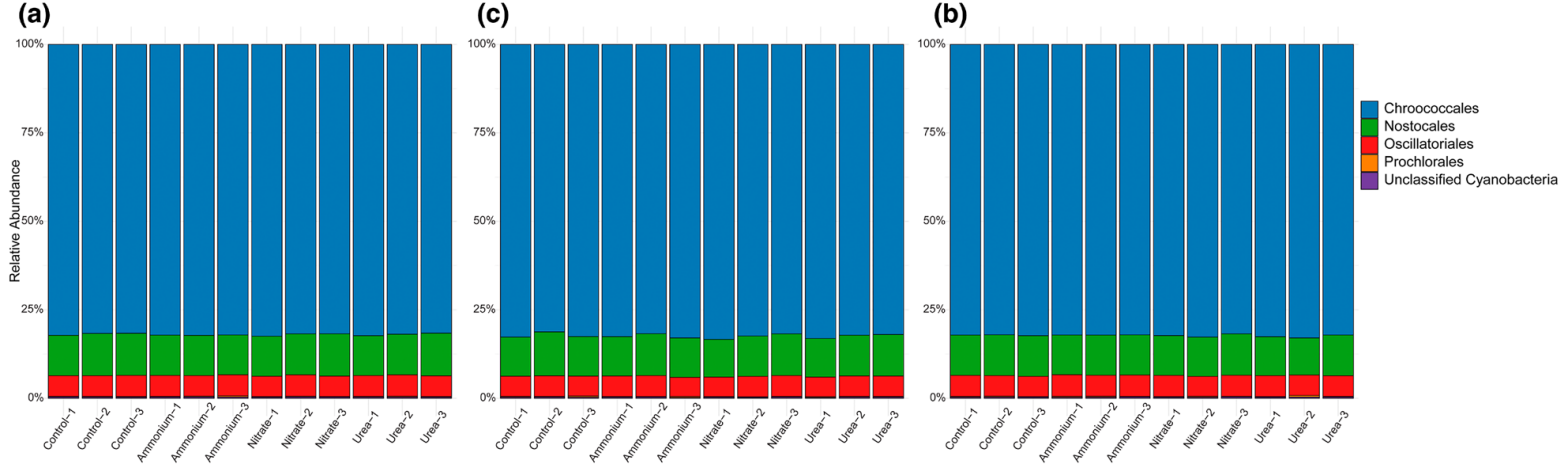
Relative abundance of major cyanobacterial taxa before nutrient additions (a), 2 h after additions (b), and 24 h after additions (c). Relative abundance calculated based on sequence libraries.

Analysis of sequence libraries shows that mesocosm communities were dominated by members of the Cyanobacteria phylum, which constituted >70% of bacterial reads at the start of the experiment (Figure 1a) with the exception of one nitrate‐treated mesocosm, which had an unusually large representation of Firmicutes. The next‐most abundant phylum was Proteobacteria, which comprised between 7% and 18% of the transcriptionally active bacterial community, followed by Firmicutes (<1%–3%, excluding the outlier), other taxa (<1%–9%), and Planctomycetes (<1%–2%; see Table S3 for a full list of taxa). The dominance of Cyanobacteria was consistent across all treatments throughout the 24‐h time series, including the control. Within that phylum, the transcriptionally active Cyanobacterial community structure was quite stable within and between treatments, with Chroococcales (including *Microcystis* spp.) comprising ~82% of the community, followed by Nostocales at ~12%, Oscillatoriales at ~6%, Prochlorales at <1%, and other unclassified Cyanobacteria at <0.5% (Figure 2).

**FIGURE 3:**
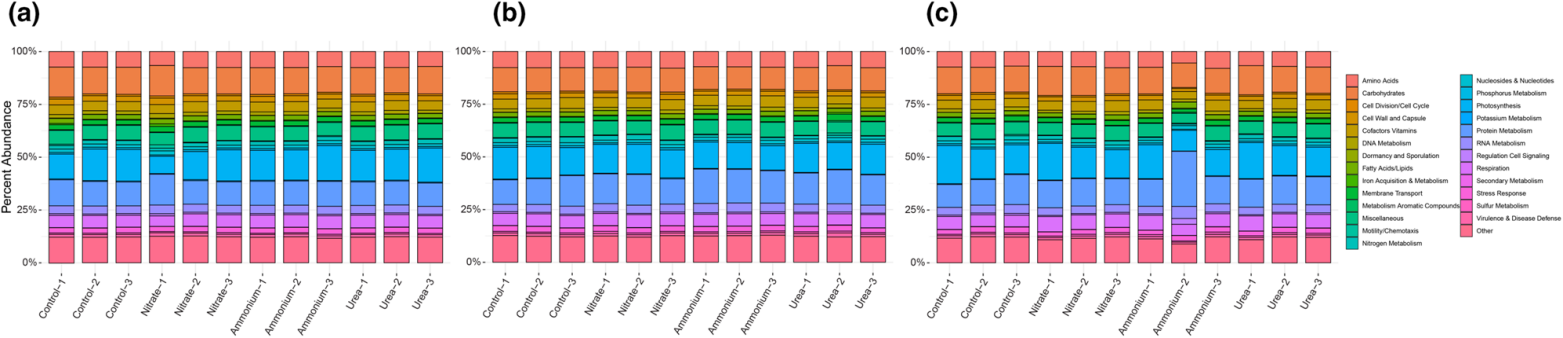
Relative proportion of bacterial function based on sequence libraries before nutrient additions (a), 2 h after nutrient additions (b), and 24 h after nutrient additions (c).

Similar to the stability of community structure according to the metatranscriptomes, active biological functions of the community sequences were comparable among treatments throughout the 24‐h time series (Figure 3). Among the various biological functions, photosynthesis (8%–18%), protein metabolism (10%–26%), and carbohydrate (10%–14%) constituted most of the community functions. Results from Kruskal–Wallis tests showed no significant effect of nitrogen additions on the biological functions of community sequences ($\chi_{3}^{2}$ ≤ 6.69; *p* ≥ 0.08).

### Global *Microcystis* gene expression

Despite the stable community structure and overall community functional profile, the transcriptional response of *Microcystis* incubated with different nitrogen forms varied significantly among treatments. All reported genes were control‐corrected, meaning that any significant change in gene regulation observed in the control was not considered a significant change in gene regulation in the nitrogen‐addition treatments. Gene activity that was inverse to the control was considered a significant change in regulation (e.g., upregulated in control, downregulated in nitrate). When compared to before the nitrogen additions, enclosures given ammonium amendments had 4.8% (232) of the total number of genes significantly upregulated and 5.8% (277) significantly downregulated after 2 h (Figure S1). Meanwhile, 2.47% (118) of genes were upregulated and 1.78% (85) were downregulated in urea‐treated enclosures after 2 h, whereas 0.97% (46) of genes were upregulated and 1.19% (57) were downregulated in nitrate‐treated enclosures after 2 h.

Significant transcriptional responses continued up to 24 h after nitrogen amendment, particularly in nitrate‐treated enclosures which had 3.4% (165) of genes upregulated and 1.7% (82) downregulated (Figure S2); more than double the number of significantly expressed genes after 2 h for this treatment. Ammonium‐treated enclosures had 3.8% (219) of genes upregulated and 3.0% (169) downregulated after 24 h. Urea‐treated enclosures had 2.4% (118) of genes upregulated and 1.3% (64) downregulated.

### Core metabolite‐related expression

**FIGURE 4:**
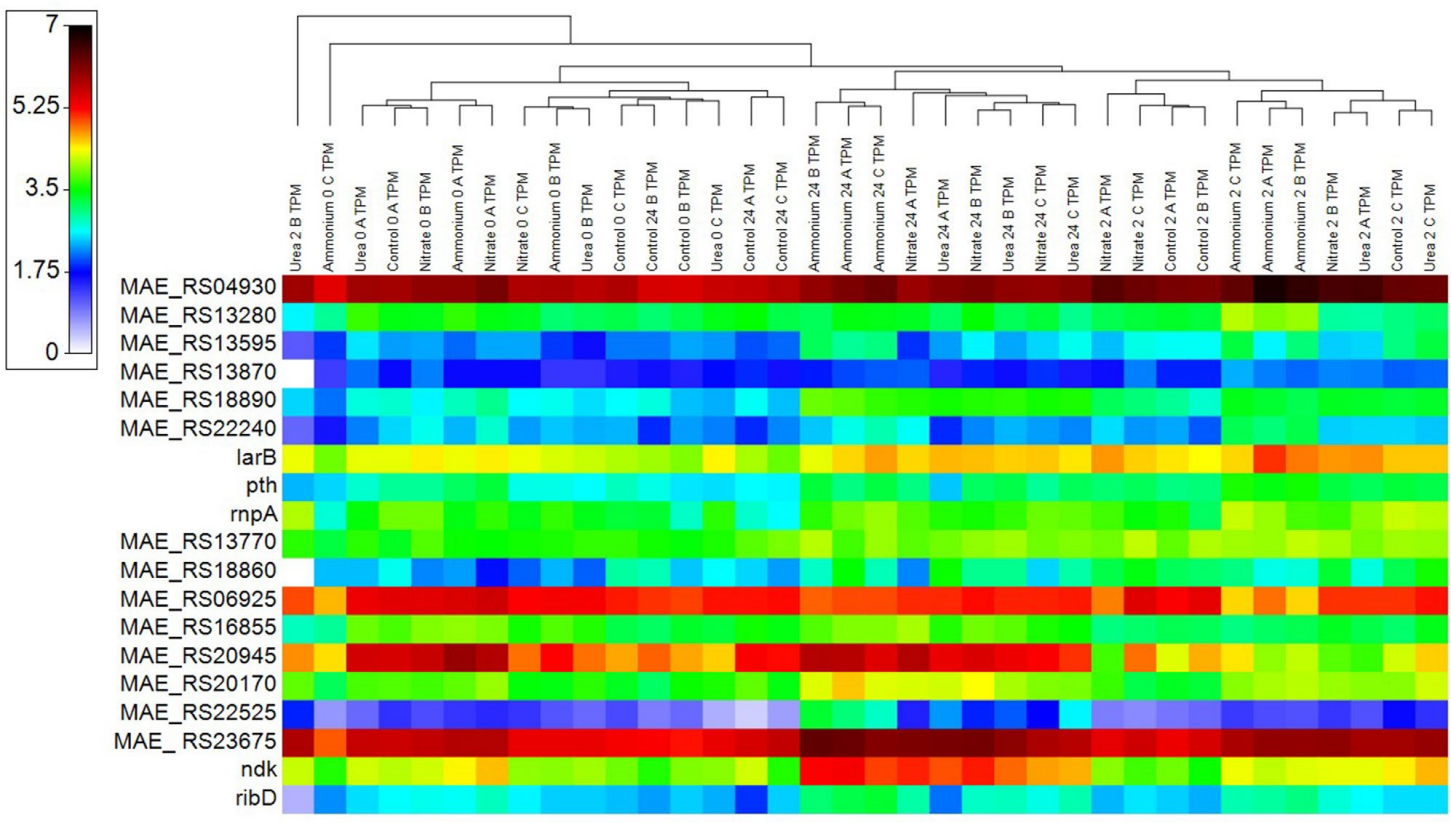
Heat map for the expression (log[x + 1] TPM values) of core metabolite associated genes of *Microcystis* sp. Dendrogram displaying the clustering of individual samples (*n* = 36) was determined using a Bray–Curtis similarity analysis. Each sample is unique to a specific enclosure and sampling time point (treatment, time point sampled, replicate).

The different nitrogen treatments had a pronounced effect on the expression of genes related to amino acids, nucleotides, and sugars in the *Microcystis* bloom communities manipulated in this experiment (Figure 4). The ammonium‐treated communities had greater upregulation of genes associated with core metabolites including sugar transferase (MAE_RS04930), pyridoxal phosphate‐dependent aminotransferase (MAE_RS13280), phosphoribosylaminoimidazolesuccinocarboxamide (SAICAR) synthase (MAE_RS13595), amino acid ABC transporter permease (MAE_RS13870), ribonucleoside‐diphosphate reductase subunit alpha (MAE_RS18890), CCA tRNA nucleotidyltransferase (MAE_RS22240), 1‐(5‐phosphoribosyl)‐5‐amino‐4‐imidazole‐carboxylate carboxylase (*lar*B), aminoacyl‐tRNA hydrolase (*pth*), ribonuclease P protein component (*rnp*A) 2 h after ammonium additions. The nitrate treatment exhibited upregulation of aspartate aminotransferase family protein (MAE_RS13770), ribonucleoside‐diphosphate reductase (MAE_RS18860), and MAE_RS18890. The urea‐treated enclosures upregulated MAE_RS04930 and MAE_RS18890. Similarly, enclosures treated with ammonium exhibited the downregulation of core metabolite genes including aspartate aminotransferase family protein (MAE_RS06925), four‐carbon acid sugar kinase family protein (MAE_RS16855), and branched‐chain amino acid ABC transporter substrate‐binding protein (MAE_RS20945) followed by nitrate, which downregulated MAE_RS20945.

After 24 h, there was a similar difference among treatments with respect to core metabolite‐associated gene expression. Ammonium‐treated communities of *Microcystis* continued to upregulate core metabolite‐related genes MAE_RS18890, MAE_13595, LL‐diaminopimelate aminotransferase (MAE_RS20170), aminotransferase class V‐fold PLP‐dependent enzyme (MAE_RS22525), TatD family deoxyribonuclease (MAE_RS23675), nucleoside‐diphosphate kinase (*ndk*), 5‐amino‐6‐(5‐phosphoribosylamino)uracil reductase (*rib*D). Nitrate‐treated communities had comparably less upregulation of core metabolite‐related genes, with MAE_RS1889 and *ndk.* Similarly, the urea‐treated communities also had fewer instances of upregulation with MAE_RS18890, MAE_RS22525, and *ndk* being the only core metabolite‐related genes to have significant upregulation.

### Restriction modification

Multiple genes involved in restriction modification (RM) systems were differentially regulated by treatment with the different nitrogen forms. At 2 h, Uma2 family endonucleases (a diverse grouping of genes associated with nucleic acid metabolism) were significantly upregulated in the ammonium (MAE_RS01080; MAE_RS04615) and nitrate (MAE_RS03170) treatments. However, a higher number of RM genes were significantly downregulated with the addition of ammonium, including a group of genes associated with the Uma2 endonuclease family (MAE_RS04820; MAE_RS07350; MAE_RS07355; MAE_RS27025) as well as the HNH endonucleases (MAE_RS26995). The nitrate‐treated communities exhibited significant increases in other genes associated with the Uma2 endonuclease family (MAE_RS15155; MAE_RS27025) and the HNH endonucleases (MAE_RS08715). The urea treatment only exhibited a significant increase in MAE_RS27025. After 24 h, ammonium‐treated communities of *Microcystis* continued to upregulate a gene associated with the Uma2 family endonuclease family (MAE_RS06250), although this differed from the Uma2 endonucleases upregulated at 2 h in the ammonium treatment (MAE_RS01080; MAE_RS04615). Downregulation of genes within the Uma2 endonucleases family continued at 24 h in all three treatments, with multiple genes downregulated by *Microcystis* exposed to elevated ammonium (MAE_RS01670; MAE_RS04820), nitrate (MAE_RS04820; MAE_RS05965), and urea (MAE_RS10470).

### Carbon acquisition and metabolism

**FIGURE 5:**
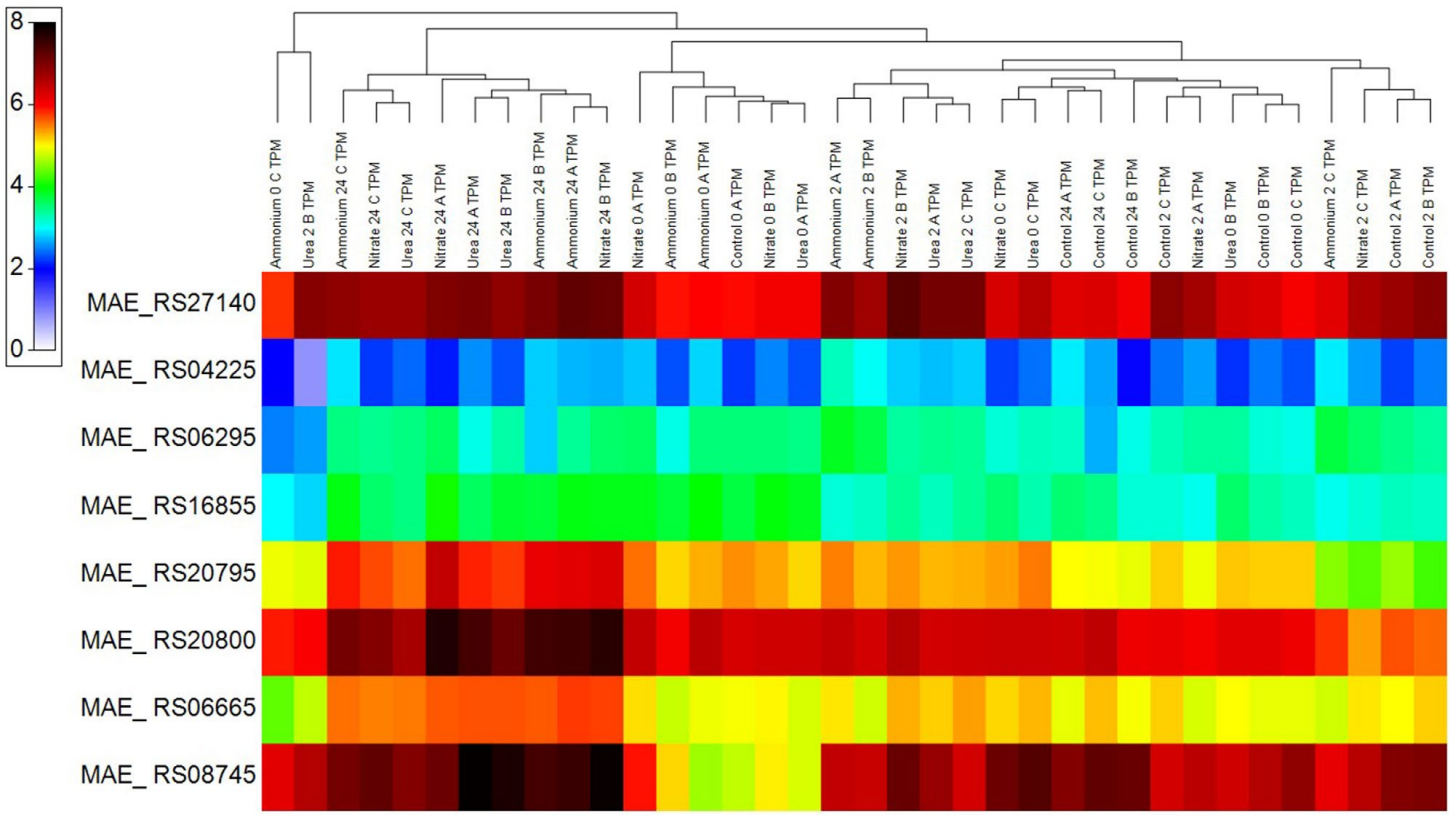
Heat map for the expression (log[x + 1]) of carbon acquisition and metabolism genes of *Microcystis* sp. Dendrogram displaying the clustering of individual samples (*n* = 36) was determined using a Bray–Curtis similarity analysis. Each sample is unique to a specific enclosure and sampling time point (treatment, time point sampled, replicate).

*Microcystis* communities had varying responses to nitrogen additions with respect to genes related to carbon acquisition and metabolism (Figure 5). Ammonium‐, urea‐, and nitrate‐treated communities had significantly higher transcription levels of bicarbonate transporters (MAE_RS27140) 2 h after nitrogen additions (*p* < 0.001). Furthermore, ammonium‐treated communities also had greater transcription of carbon storage regulator (MAE_RS04225) and carbonic anhydrase (MAE_RS06295) and decreased transcription of the four‐carbon acid sugar kinase family protein (MAE_RS16855) 2 h after treatment.

A notable shift in carbon‐related gene transcription occurred in nitrate‐ and ammonium‐treated communities, for which carbon‐concentrating mechanism genes (MAE_RS20795, MAE_RS20800), carbonate dehydratase (MAE_RS06665), and bicarbonate transport system proteins (MAE_RS08745) had greater transcription compared to before nitrogen additions (*p* < 0.001). After 24 h, *Microcystis* communities treated with urea had no significant expression of carbon‐related genes (*p* > 0.05).

### Nitrogen acquisition and metabolism

*Microcystis* communities had notable and somewhat expected responses to nitrogen additions with respect to expression of genes related to nitrogen acquisition and metabolism (Figure S3). Nitrate‐treated enclosures had significant upregulation of nitrate ABC transporter permease (ntrB_1) and nitrate reductase (MAE_RS23440) 2 h after treatment. Ammonium‐treated enclosures upregulated pyroglutamyl‐peptidase (MAE_RS13070). Meanwhile, urea‐treated enclosures had no significant upregulation of nitrogen‐related genes 2 h after treatment. All *Microcystis* communities had significant downregulatory responses to several key nitrogen‐related genes 2 h after treatment. Nitrate‐treated communities had significant downregulation of urea ABC transporter permease (urtC). Ammonium‐treated enclosures had significant downregulation of the nblA protein (MAE_RS01135; a key protein in pigment degradation and signal for nitrogen starvation), urea ABC transporter ATP‐binding subunit (urtE), urea ABC transporter ATP‐binding protein (urtD), urtC, urea ABC transporter permease subunit (*urt*B), urea ABC transporter substrate‐binding protein (urtA), ntrB_1, nitrate ABC transporter substrate‐binding protein (MAE_RS06515), ferredoxin‐nitrite reductase (MAE_RS08065), 2‐nitropropane dioxygenase (MAE_RS12145), MAE_RS23440, type I glutamate‐ammonia ligase (glnA), glutamine synthetase (MAE_RS03995), acetylglutamate kinase (argB), and transglutaminase family protein (MAE_RS14510). The urea‐treated *Microcystis* communities exhibited significant downregulation of nblA protein (MAE_RS01135), urtC, urtB, ntrB_1, MAE_RS12145, MAE_RS03995, and MAE_RS14510.

The nitrogen‐treated *Microcystis* communities exhibited a notable shift in upregulated genes 24 h after nitrogen additions. Nitrate‐treated communities upregulated MAE_RS06515, nitrate ABC transporter permease protein (*ntr*B_2), MAE_RS08065, and MAE_RS23440. Ammonium‐treated communities upregulated *ntr*B_2. Interestingly, all communities also upregulated key genes related to pigment synthesis (Figure 4). The nitrate‐treated enclosures upregulated phycobilisome rod‐core linker polypeptide CpcG (MAE_RS21010), allophycocyanin subunit beta (*apc*B_1), allophycocyanin subunit alpha (MAE_RS04495), phycocyanin subunit beta (MAE_RS10675), phycocyanin subunit alpha (*cpc*A_1), phycocyanin subunit alpha (*cpc*A_2), and phycocyanin subunit beta (MAE_RS22455). The ammonium treatment upregulated *apc*B*_*1, MAE_RS04495, MAE_RS10675, *cpc*A_1, and *cpc*A*_*2. Meanwhile, the urea‐treated enclosures upregulated *cpc*A*_*2 and MAE_RS22455.

Lastly, *Microcystis* communities exhibited significant downregulation of several key genes related to nitrogen acquisition and metabolism 24 h after treatment. Ammonium‐treated *Microcystis* downregulated MAE_RS01135, *urt*E, *urt*D, *urt*C, *urt*B, *urt*A, *ntr*B_1, MAE_RS06515, MAE_RS08065, MAE_RS23440, transglutaminase (MAE_RS02445), MAE_RS03995, *arg*B, and MAE_RS14510. Nitrate‐treated communities downregulated MAE_RS01135, *urt*C, and *urt*B. Urea‐treated communities downregulated MAE_RS01135, *urt*E, *urt*D, *urt*C, *urt*B, and *arg*B.

### *mcy* gene cluster expression

**FIGURE 6:**
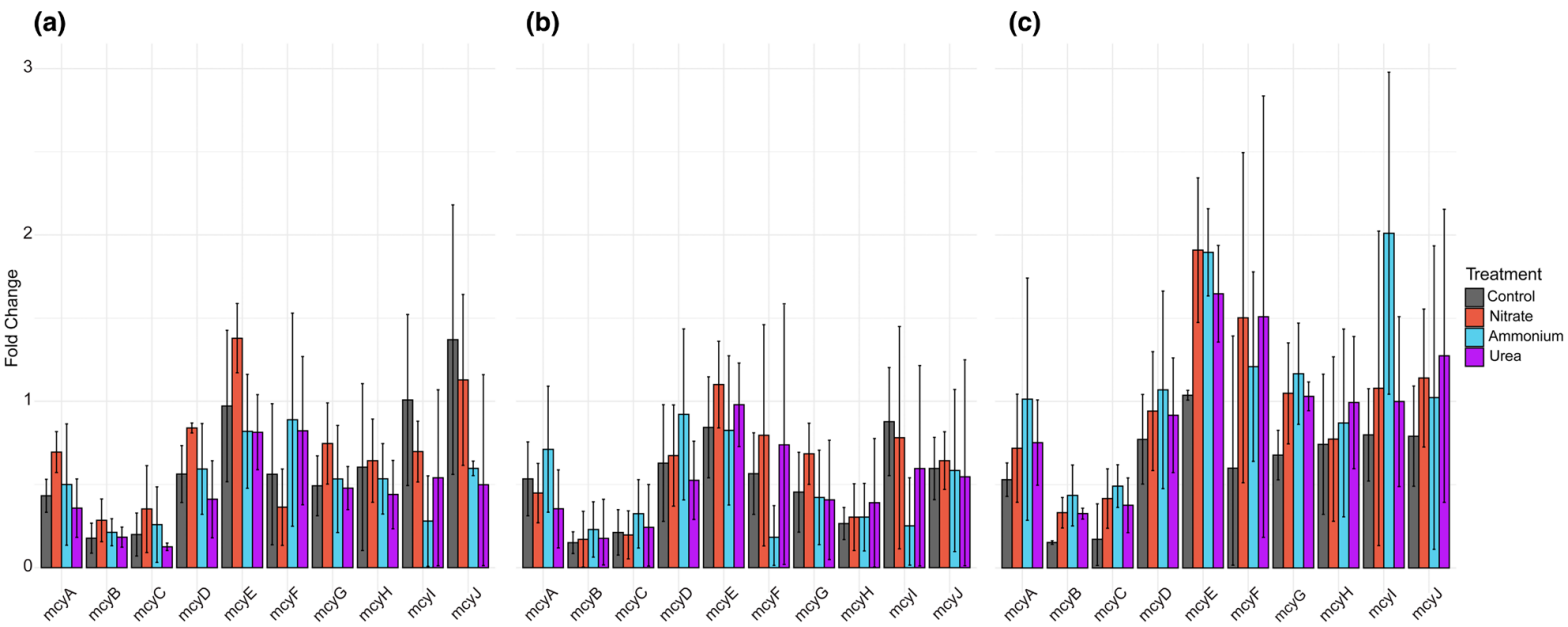
Comparison of fold change values of the *mcy* gene cassette between the different treatments before nutrient additions (a), 2 h after nutrient additions (b), and 24 h after nutrient additions (c).

Genes related to production of the toxin microcystin were examined to uncover any trends to help the understanding of if/how nutrients, specifically nitrogen, influence their expression. The entire *mcy* toxin gene cassette (*mcy*ABCDEFGHIJ) was transcribed in each treatment during the experiment (Figure 6). No effect of nitrogen amendments was detected during statistical analyses; however, *mcy*E (*p* = 0.0001; *F*_2,16_ = 17.5275; repeated‐measures analysis of variance, RM‐ANOVA), *mcy*G (*p* = 0.0003; *F*_2,16_ = 13.8297; RM‐ANOVA), *mcy*H (*p* = 0.0079; *F*_2,16_ = 6.6501; RM‐ANOVA), and *mcy*I (*p* = 0.0300; *F*_2,16_ = 4.4031; RM‐ANOVA) all had significant increases in expression within treatments after 24 h. No significant differences in the expression of the genes within the *mcy* toxin gene cassette were observed within the control group. Lastly, a marginally insignificant effect of nitrogen on the expression of *mcy*E was observed (*p* = 0.0501; *F*_3,8_ = 4.0610; RM‐ANOVA). The post hoc test identified significantly greater expression in ammonium (*p* = 0.0408) and nitrate (*p* = 0.0380) enclosures compared to the control 24 h after additions (Figure 6).

## DISCUSSION

Blooms of *Microcystis* and most other Cyanobacterial taxa are global issues that threaten valuable freshwater resources and as such have attracted the focus of research teams for decades. Understanding how these organisms respond to the ever‐increasing concentrations of reduced nitrogen forms, like ammonium and urea (see Glibert et al., 2006, 2016), is critical for mitigation of future bloom events. Specifically, understanding how *Microcystis* and the phycosphere as a whole respond on the transcriptional level in a field setting is particularly useful. This experiment monitored the response of *Microcystis* and the rest of the bacterial members of the phycosphere over a 24‐h period following the addition of three common nitrogen forms, representative of a pulse of nutrients similar to what would occur as runoff after a storm event.

### Global *Microcystis* gene expression

*Microcystis* gene expression was quite variable depending on the form of nitrogen added to the enclosures demonstrating that *Microcystis* transcriptional responses are, in part, determined by the dominant nitrogen form. Only 2 h after additions, ammonium‐treated enclosures had the greatest difference in gene expression (509 genes, 10.6%) followed by urea (203 genes, 4.3%) and nitrate (103 genes, 2.2%). However, there was a distinct shift 24 h after nitrogen additions in which nitrate enclosures more than doubled the number of significantly expressed genes compared to the 0 h time point (247 genes, 5.1%), while ammonium (388 genes, 6.8%) and urea additions (182 genes, 3.7%) each resulted in decreases in total gene expression. The chosen study system contained approximately 2 mg · L^−1^ TN and no detectable amounts of dissolved nitrogen (NO_3_‐N, NH_3_+NH_4_, and urea) prior to the start of the experiment, although it is unclear for how long the *Microcystis* could be considered nutrient starved. These conditions appear to be slightly unique when compared to other field experiments conducted in larger sites that often had measurable amounts of dissolved nitrogen during the project such as Lake Erie (between 312.96 and 954.96 μg · L^−1^, Chaffin & Bridgeman, 2014; between 77.95 and 1361.63 μg · L^−1^, Chaffin et al., 2018) and Wascana Lake (between 1200 and 1600 μg · L^−1^, Donald et al., 2011). However, Li et al. (2023) observed similarly low TDN values prior to the onset of harmful cyanobacterial blooms in Lake Utah (~31 μg · L^−1^), which were even lower after the bloom had senesced (~11 μg · L^−1^).

Ammonium is generally recognized as a more energetically efficient nitrogen source than nitrate (Flores & Herrero, 2005; Herrero et al., 2001; Krausfeldt et al., 2020). This understanding may explain why ammonium‐treated *Micrcocystis* communities appear to have a more rapid and pronounced transcriptional response compared to nitrate. However, urea‐treated communities had a comparatively lower transcriptional response to additions, while also having comparable amounts of pigments and total number of cyanobacterial cells to ammonium‐treated enclosures (see Gladfelter et al., 2022). Culture experiments such as that by Krausfeldt et al. (2020) reported similar findings, and the authors postulated that the role of urea as both a carbon and nitrogen source may allow for *Microcystis* to overcome certain rate‐limiting steps, thus expressing fewer genes, to produce the necessary metabolic products (Flores & Herrero, 2005; Herrero et al., 2001; Muro‐Pastor et al., 2005; Tandeau de Marsac et al., 2001). The comparatively low transcriptional response from nitrate‐treated communities could be a result of typical cyanobacterial gene expression in which a greater response should be expected in the presence of excess ammonium, which will exert a repressive response toward genes associated with alternative nitrogen forms (Luque et al., 1994; Ohashi et al., 2011). Therefore, during this experiment, *Microcystis* cells were already expressing genes associated with uptake and assimilation of alternative nitrogen sources resulting in additions of nitrate eliciting a less significant response than those from additions of ammonium (Luque et al., 1994; Ohashi et al., 2011).

In this experiment, we observed significant changes in gene expression following the addition, rather than starvation, of nitrogen. Previous culture experiments have examined *Microcystis* transcriptional responses in nitrogen‐depleted environments, but there is limited evidence for how the phycosphere responds to introductions of different nitrogen forms in the field. Other lab‐based experiments have also reported large differences in gene expression according to the nitrogen source (Harke & Gobler, 2013, 2015; Steffen et al., 2014; Zhou et al., 2020). Steffen et al. (2014) grew *Microcystis* cultures under low phosphorus, low ammonium as the nitrogen source, low urea as the nitrogen source, and low nitrate as the nitrogen source and found similarly large differences in gene expression. In their study, the authors found the greatest difference in gene expression in cultures grown with urea as the nitrogen source (~15%), followed by ammonium (~3.5%) and nitrate (~1.7%). Furthermore, a culture experiment by Krausfeldt et al. (2020) found differential expression of glutamate‐ and glutamine‐related genes in *Microcystis* when grown with ammonium, nitrate, or urea as the nitrogen source. The authors found glutamine and glutamate followed a clear diel pattern when grown with ammonium or nitrate as the nitrogen source whereas cells grown on urea showed no such fluctuations. Conversely, their results also showed that ammonium and urea‐treated cultures had higher amounts of AMP/dGMP relative to cultures grown on nitrate, highlighting the complexity of *Microcystis* response to these nitrogen forms. The results from this field‐based experiment seem to suggest that inputs of excess ammonium cause a rapid and high transcriptional response. Future studies should continue to examine how the entire phycosphere responds to different levels and forms of nutrients (nitrogen and phosphorus), especially in the field, including in systems of varying sizes and depths as these factors likely influence the extent of biotic and abiotic exchange between the sediment and surface waters which the bloom resides (Li et al., 2024).

### Carbon metabolism

The form of nitrogen added to *Microcystis* communities had the expected effect on the expression of genes associated with carbon acquisition and metabolism. Two hours after addition, ammonium‐treated communities seemed to be the only ones expressing genes linked to carbon regulation. However, after 24 h, ammonium‐ and nitrate‐treated communities had significantly increased transcription of carbon concentrating genes (MAE_RS20795, MAE_RS20800, MAE_RS20805) and bicarbonate transport genes (MAE_RS08745, MAE_RS27140), whereas urea‐treated communities had no such expression. This would suggest that *Microcystis* cells in the ammonium and nitrate treatments were actively importing and processing extracellular carbon while cells using urea as a nitrogen source were not.

Results from this field experiment align with the findings of Krausfeldt et al. (2019) who found that *Microcystis aeruginosa* cultures were capable of using urea as both a nitrogen and a carbon source. Enclosures from this experiment that were supplemented with nitrate and ammonium had significant upregulation in genes related to carbon concentration and acquisition only 24 h after additions. Meanwhile, urea‐treated enclosures showed no such expression in carbon acquisition or concentration mechanisms. Such findings suggest that urea could further encourage and sustain intense cyanobacterial growth without the impact of carbon limitation at elevated pH. This becomes even more troublesome, as urea is perhaps the most widely used agricultural fertilizer worldwide (Glibert et al., 2006, 2014). Future studies should continue to examine how urea impacts cyanobacterial communities in the field.

### Nitrogen metabolism

*Microcystis* communities had a largely expected response to nitrogen additions in respect to the regulation of nitrogen‐related genes. *Microcystis* in nitrate‐treated enclosures had the most upregulation after 2 h, upregulating the genes for necessary nitrate transporters and reductases (*ntrB*_1, MAE_RS23440). Meanwhile, ammonium‐ and urea‐treated enclosures had no major responses. This is likely because ammonium can passively diffuse into cyanobacterial cells (Boussiba & Gibson, 1991; Muro‐Pastor & Florencio, 2003) and/or because organic nitrogen forms may have been the dominant nitrogen form prior to nitrogen additions (total dissolved nitrogen prior to start of experiment was undetectable, but experiment site was used for catfish aquaculture) meaning that importing organic nitrogen would not be quantified as significant upregulation. Future experiments should examine how the form of nitrogen used to grow a cyanobacterial community influences its gene response to new and different nitrogen sources.

At 24 h, the *Microcystis* in nitrate‐treated enclosures again had the most nitrogen‐related gene upregulation as the cells continued to upregulate genes associated with nitrate assimilation (MAE_RS06515, MAE_RS08065, MAE_RS23440, *ntr*B_2). An interesting pattern that was noticed among all treatments that received nitrogen additions was the significant upregulation of numerous genes associated with pigment synthesis (MAE_RS04495, MAE_RS10675, MAE_RS21010, MAE_RS22455, *apc*B_1, *cpc*A_1, *cpc*A_2). There is a growing body of evidence that suggests that cyanobacteria, such as *Microcystis,* are capable of storing excess amounts of nitrogen as accessory pigments, such as phycocyanin, for later use during nitrogen stress (Chaffin & Bridgeman, 2014; Elmorjani & Herdman, 1987; Erratt et al., 2018; Gladfelter et al., 2022). Such a strategy would allow cyanobacterial blooms to persist for longer, thus extending the harmful secondary effects of bloom events (anoxia, toxins, off‐flavors) and should be further explored to better understand if certain forms or nitrogen are more easily stored and/or how long cells could persist on the stored nitrogen from accessory pigments.

### Toxin production

Cyanobacterial toxins can be acutely harmful in high concentrations and have garnered much attention in recent decades due to their threat to wildlife, livestock, and humans. The toxin of focus for this experiment, microcystin, is a hepatotoxin and potential carcinogen produced by several cyanobacterial taxa, including the *Microcystis* that dominated the experimental site and enclosures. Countless experiments, studies, and reviews have looked to identify the conditions that promote the production of microcystin and other cyanobacterial toxins (Buley et al., 2022; Gągała et al., 2014; Henao et al., 2020; Holland & Kinnear, 2013; Kaebernick & Neilan, 2001; Paerl & Huisman, 2008; Paerl & Otten, 2013). Among the commonly reported important environmental triggers for toxin production, temperature, total nitrogen, total phosphorus, dissolved nitrogen, dissolved phosphorus, pH, and even dissolved organic carbon, there is little consensus. There is increasing evidence that suggests nutrient stress and/or oxidative stress is a greater influence on toxin production in cyanobacteria (Ginn et al., 2010; Krausfeldt et al., 2020; Kurmayer, 2011; Oh et al., 2000; Pimentel & Giani, 2014; Rzymski et al., 2020; Xie et al., 2016). Buley et al. (2022) synthesized environmental data from 2643 waterbodies and found a significant negative correlation between microcystin concentration and total dissolved nitrogen. Nitrogen additions in this experiment had a weak effect on *mcy* gene cluster expression. The *mcy*E gene was the only gene to have marginal treatment effects and/or differences in expression from the control. Any other differences observed were an effect of time rather than the nitrogen forms added. This could be a result of the complexities associated with field‐based experiments and relative lack of understanding as to the triggers of toxin‐related gene expression. Further research is still required to truly uncover when and why cyanobacterial cells produce toxins.

### Limited bacterial response to nitrogen additions

Although *Microcystis* showed a rapid response to the various nitrogen additions, the co‐occurring bacterial community was comparably resistant to the pulse additions. This lack of treatment effect extends to the active biological functions of community sequences as well. These observations are consistent with previous field experiments. Gobler and Jankowiak (2022) examined the response of the endosymbiotic microbial communities associated with *Microcystis* colonies from lakes within the Laurentian Great Lakes region by subjecting the *Microcystis* colonies to elevated amounts of nitrogen, phosphorus, and/or temperature. The authors found that the co‐occurring bacteria were resistant to the applied treatments. This extended to measurements including beta diversity and predicted functional potential. They would go on to conclude that cyanobacterial abundance (i.e., bloom intensity) had a greater effect on the composition and function of the co‐occurring bacterial community. These results are further in line with previous studies including Jankowiak and Gobler (2020) who sampled the microbiomes of *Microcystis* colonies within two different lakes and found that the functional potential of the different microbiomes was largely conserved despite the spatial separation. Furthermore, the authors also suggested bloom intensity to be the major source of selection pressure on the co‐occurring microbiota. These results corroborate what was observed in the present study and highlight the need for further investigation into how the co‐occurring microbial community manages environmental stimuli to limit substantial changes.

## AUTHOR CONTRIBUTIONS

**Matthew F. Gladfelter:** Conceptualization (equal); data curation (equal); formal analysis (equal); investigation (equal); methodology (equal); visualization (equal); writing – original draft (equal); writing – review and editing (equal). **Hunter R. Baylous:** Data curation (equal); investigation (equal); methodology (equal); writing – review and editing (equal). **Alan E. Wilson:** Conceptualization (equal); funding acquisition (equal); resources (equal); supervision (equal); validation (equal); writing – review and editing (equal). **Morgan M. Steffen:** Conceptualization (equal); funding acquisition (equal); resources (equal); supervision (equal); writing – review and editing (equal).

## FUNDING INFORMATION

This project was supported through USDA NIFA 2017‐70007‐27132, USDA‐ARS 58‐6010‐0‐006, NSF DEB‐1831094, the Alabama Agricultural Experiment Station, and the Hatch program of the National Institute of Food and Agriculture, U.S. Department of Agriculture. We would like to thank these funding providers for allowing us to conduct this research. M.M.S. and H.R.B. were supported by NSF DEB‐1831106.

## DECLARATION OF GENERATIVE AI IN SCIENTIFIC WRITING

All authors declare that no generative AI was used at any point throughout the development, implementation, collection, analysis, or writing of any material associated with this research project.

## Supporting information


**Table S1.** Coverage of each library. Coverage was calculated based on the number of bp that mapped to the *M. aeruginosa* NIES 843 genome (5,842,795 bp) during expression analysis.
**Table S2.** Hits per sample broken down by domain, according to analysis of short‐read libraries done in MG‐RAST.
**Table S3.** Hits per sample broken down by phylum, according to analysis of short‐read libraries done in MG‐RAST.
**Figure S1.** Scatter plots for the nutrient treatments comparing the 0 h gene expression values to the 2 h gene expression values. Green dots are genes that were significantly upregulated, red dots are genes that were significantly downregulated, and gray dots are genes that had no significant change in gene expression.
**Figure S2.** Scatter plots for the nutrient treatments comparing the 0 h gene expression values to the 24 h gene expression values. Green dots are genes that were significantly upregulated, red dots are genes that were significantly downregulated, and gray dots are genes that had no significant change in gene expression.
**Figure S3.** Heat map for the expression (log[x + 1]) of nitrogen acquisition and metabolism genes of *Microcystis* sp. Dendrogram displaying the clustering of individual samples (*n* = 36) was determined using a Bray–Curtis similarity analysis. Each sample is unique to a specific enclosure and sampling time point (Treatment, time point sampled, replicate).
**Figure S4.** Heat map for the expression (log[x + 1]) of pigment associated genes of *Microcystis* sp. Dendrogram displaying the clustering of individual samples (*n* = 36) was determined using a Bray–Curtis similarity analysis. Each sample is unique to a specific enclosure and sampling time point (Treatment, time point sampled, replicate).

## Data Availability

Data from this experiment are available upon serious request to the corresponding author. Sequencing data associated with this experiment can be accessed through NCBI's Sequence Read Archive (SRA) after October 31, 2026 using the accession number PRJNA1337457 or by using the following link: https://www.ncbi.nlm.nih.gov/sra/PRJNA1337457.
