# Supplementary material for: Nutrient disturbance in a shallow aquaculture pond impacts Microcystis gene expression but does not impact bacterial community function during bloom conditions
Source: J Phycol. 2026 Jul 9;62(4):1297–310. doi: 10.1111/jpy.70197 (PMC13485200; doi:10.1111/jpy.70197)
Supplement: Supplementary file 1 [file JPY-62-1297-s001.docx]

Nutrient disturbance in a shallow aquaculture pond impacts *Microcystis* gene expression but does not impact bacterial community function during bloom conditions

Matthew F. Gladfelter^1^, Hunter R. Baylous^2^, and Alan E. Wilson^1^, Morgan M. Steffen^2^*

^1^Auburn University School of Fisheries, Aquaculture, and Aquatic Sciences, Auburn, AL, USA

^2^James Madison University Department of Biology, Harrisonburg, VA, USA

*corresponding author: Morgan M. Steffen; steffemm@jmu.edu

**Table S1.** Coverage of each library. Coverage was calculated based on the number of bp that mapped to the *M. aeruginosa* NIES 843 genome (5,842,795 bp) during expression analysis.

| **Library** | **Sample** | **Base Pairs Mapped** | **Coverage** |
| --- | --- | --- | --- |
| H00E03 | Control 0 H A | 4,540,055,400 | 777x |
| H00E16 | Control 0 H B | 3,930,440,850 | 673x |
| H00E19 | Control 0 H C | 3,209,005,800 | 549x |
| H02E03 | Control 2 H A | 3,379,263,000 | 578x |
| H02E16 | Control 2 H B | 3,751,523,850 | 642x |
| H02E19 | Control 2 H C | 3,583,110,750 | 613x |
| H24E03 | Control 24 H A | 2,500,888,800 | 428x |
| H24E16 | Control 24 H B | 5,330,013,000 | 912x |
| H24E19 | Control 24 H C | 2,670,812,700 | 457x |
| H00E05 | NH4 0 H A | 3,403,194,900 | 582x |
| H00E13 | NH4 0 H B | 3,232,160,850 | 553x |
| H00E17 | NH4 0 H C | 2,179,004,250 | 373x |
| H02E05 | NH4 2 H A | 3,218,252,850 | 551x |
| H02E13 | NH4 2 H B | 3,055,930,200 | 523x |
| H02E17 | NH4 2 H C | 4,000,430,850 | 685x |
| H24E05 | NH4 24 H A | 3,966,167,700 | 679x |
| H24E13 | NH4 24 H B | 669,621,300 | 115x |
| H24E17 | NH4 24 H C | 4,956,692,850 | 848x |
| H00E08 | Urea 0 H A | 3,761,594,850 | 644x |
| H00E15 | Urea 0 H B | 3,784,563,150 | 648x |
| H00E23 | Urea 0 H C | 3,412,296,600 | 584x |
| H02E08 | Urea 2 H A | 3,226,257,600 | 552x |
| H02E15 | Urea 2 H B | 609,536,550 | 104x |
| H02E23 | Urea 2 H C | 4,292,431,200 | 735x |
| H24E08 | Urea 24 H A | 1,886,750,850 | 323x |
| H24E15 | Urea 24 H B | 5,168,680,500 | 885x |
| H24E23 | Urea 24 H C | 3,586,032,900 | 614x |
| H00E01 | NO3 0 H A | 1,958,703,300 | 335x |
| H00E11 | NO3 0 H B | 3,659,534,250 | 626x |
| H00E18 | NO3 0 H C | 4,012,601,250 | 687x |
| H02E01 | NO3 2 H A | 3,013,643,550 | 516x |
| H02E11 | NO3 2 H B | 2,918,315,700 | 499x |
| H02E18 | NO3 2 H C | 4,013,747,850 | 687x |
| H24E01 | NO3 24 H A | 2,856,035,250 | 489x |
| H24E11 | NO3 24 H B | 4,863,872,400 | 832x |
| H24E18 | NO3 24 H C | 5,701,731,600 | 976x |

**Table S2.** Hits per sample broken down by domain, according to analysis of short-read libraries done in MG-RAST

| **Enclosure** | **Interval** | **Archaea** | **Bacteria** | **Eukaryota** | | **Viruses** |
| --- | --- | --- | --- | --- | --- | --- |
| NitrateA | 0 h | 1397 | 1556144 | 391989 | 1188 | |
| ControlA | 0 h | 1313 | 980079 | 142039 | 953 | |
| AmmoniumA | 0 h | 1414 | 896531 | 175035 | 826 | |
| UreaA | 0 h | 1785 | 1077896 | 130644 | 900 | |
| NitrateB | 0 h | 1638 | 1000704 | 113559 | 798 | |
| AmmoniumB | 0 h | 968 | 668575 | 100461 | 589 | |
| UreaB | 0 h | 1082 | 728629 | 100113 | 627 | |
| ControlB | 0 h | 1288 | 822603 | 103759 | 633 | |
| AmmoniumC | 0 h | 1218 | 557138 | 93413 | 627 | |
| NitrateC | 0 h | 892 | 702862 | 88044 | 574 | |
| ControlC | 0 h | 954 | 571024 | 70236 | 512 | |
| UreaC | 0 h | 770 | 489642 | 51018 | 346 | |
| NitrateA | 2 h | 740 | 593324 | 49512 | 355 | |
| ControlA | 2 h | 874 | 671028 | 49497 | 457 | |
| AmmoniumA | 2 h | 1122 | 819814 | 93758 | 553 | |
| UreaA | 2 h | 925 | 761338 | 67069 | 475 | |
| NitrateB | 2 h | 979 | 729402 | 73163 | 440 | |
| AmmoniumB | 2 h | 1265 | 951679 | 81540 | 866 | |
| UreaB | 2 h | 1534 | 237318 | 65301 | 598 | |
| ControlB | 2 h | 1194 | 795248 | 92972 | 579 | |
| AmmoniumC | 2 h | 1324 | 1089435 | 55593 | 847 | |
| NitrateC | 2 h | 839 | 683744 | 71889 | 477 | |
| ControlC | 2 h | 850 | 763710 | 71359 | 512 | |
| UreaC | 2 h | 1089 | 884457 | 83617 | 610 | |
| NitrateA | 24 h | 858 | 662295 | 45922 | 453 | |
| ControlA | 24 h | 604 | 374604 | 63838 | 387 | |
| AmmoniumA | 24 h | 1200 | 1090819 | 66801 | 827 | |
| UreaA | 24 h | 1150 | 727043 | 72435 | 476 | |
| NitrateB | 24 h | 1492 | 1260465 | 96876 | 997 | |
| AmmoniumB | 24 h | 893 | 554225 | 2401122 | 714 | |
| UreaB | 24 h | 1693 | 1487048 | 138746 | 1075 | |
| ControlB | 24 h | 1392 | 1096660 | 147004 | 868 | |
| AmmoniumC | 24 h | 2219 | 1787428 | 93917 | 1553 | |
| NitrateC | 24 h | 2055 | 1625908 | 125650 | 1385 | |
| ControlC | 24 h | 1019 | 627391 | 106004 | 872 | |
| UreaC | 24 h | 1191 | 921909 | 127674 | 675 | |

| **Enclosure** | **Interval** | **Other** | **Sum** |
| --- | --- | --- | --- |
| NitrateA | 0 h | 4 | 1950722 |
| ControlA | 0 h | 1 | 1124385 |
| AmmoniumA | 0 h | 0 | 1073806 |
| UreaA | 0 h | 1 | 1211226 |
| NitrateB | 0 h | 1 | 1116700 |
| AmmoniumB | 0 h | 0 | 770593 |
| UreaB | 0 h | 0 | 830451 |
| ControlB | 0 h | 1 | 928284 |
| AmmoniumC | 0 h | 2 | 652398 |
| NitrateC | 0 h | 0 | 792372 |
| ControlC | 0 h | 0 | 642726 |
| UreaC | 0 h | 2 | 541778 |
| NitrateA | 2 h | 1 | 643932 |
| ControlA | 2 h | 0 | 721856 |
| AmmoniumA | 2 h | 1 | 915248 |
| UreaA | 2 h | 1 | 829808 |
| NitrateB | 2 h | 2 | 803986 |
| AmmoniumB | 2 h | 1 | 1035351 |
| UreaB | 2 h | 6 | 304757 |
| ControlB | 2 h | 1 | 889994 |
| AmmoniumC | 2 h | 1 | 1147200 |
| NitrateC | 2 h | 0 | 756949 |
| ControlC | 2 h | 1 | 836432 |
| UreaC | 2 h | 0 | 969773 |
| NitrateA | 24 h | 1 | 709529 |
| ControlA | 24 h | 7 | 439440 |
| AmmoniumA | 24 h | 0 | 1159647 |
| UreaA | 24 h | 2 | 801106 |
| NitrateB | 24 h | 4 | 1359834 |
| AmmoniumB | 24 h | 39 | 2956993 |
| UreaB | 24 h | 1 | 1628563 |
| ControlB | 24 h | 2 | 1245926 |
| AmmoniumC | 24 h | 3 | 1885120 |
| NitrateC | 24 h | 5 | 1755003 |
| ControlC | 24 h | 0 | 735286 |
| UreaC | 24 h | 0 | 1051449 |

**Table S3.** Hits per sample broken down by phylum, according to analysis of short-read libraries done in MG-RAST

| **Enclosure** | **Interval** | **Acidobacteria** | **Actinobacteria** | **Aquificae** | **Bacteroidetes** |
| --- | --- | --- | --- | --- | --- |
| NitrateA | 0 h | 3123 | 9770 | 148 | 38 |
| ControlA | 0 h | 2895 | 10495 | 188 | 17554 |
| AmmoniumA | 0 h | 3536 | 12414 | 208 | 19819 |
| UreaA | 0 h | 4985 | 14454 | 315 | 76 |
| NitrateB | 0 h | 3718 | 9932 | 165 | 51 |
| AmmoniumB | 0 h | 2478 | 9133 | 148 | 11723 |
| UreaB | 0 h | 2559 | 9876 | 203 | 32 |
| ControlB | 0 h | 3578 | 14443 | 279 | 15575 |
| AmmoniumC | 0 h | 2767 | 21704 | 267 | 39 |
| NitrateC | 0 h | 2530 | 6897 | 150 | 37 |
| ControlC | 0 h | 1973 | 5875 | 135 | 19 |
| UreaC | 0 h | 1636 | 4974 | 95 | 23 |
| NitrateA | 2 h | 1953 | 4906 | 154 | 9937 |
| ControlA | 2 h | 2233 | 4359 | 105 | 17 |
| AmmoniumA | 2 h | 2231 | 14250 | 237 | 28 |
| UreaA | 2 h | 2144 | 6422 | 129 | 33 |
| NitrateB | 2 h | 2593 | 12757 | 250 | 8961 |
| AmmoniumB | 2 h | 2261 | 17759 | 235 | 11014 |
| UreaB | 2 h | 1234 | 21398 | 0 | 192 |
| ControlB | 2 h | 3259 | 8288 | 216 | 38 |
| AmmoniumC | 2 h | 2534 | 11544 | 292 | 22033 |
| NitrateC | 2 h | 1914 | 5118 | 98 | 12 |
| ControlC | 2 h | 2158 | 5769 | 129 | 9413 |
| UreaC | 2 h | 2526 | 10088 | 163 | 9372 |
| NitrateA | 24 h | 1370 | 6685 | 97 | 5573 |
| ControlA | 24 h | 1079 | 12354 | 78 | 5748 |
| AmmoniumA | 24 h | 2143 | 10034 | 191 | 11602 |
| UreaA | 24 h | 1497 | 8984 | 152 | 23 |
| NitrateB | 24 h | 3139 | 13380 | 272 | 35 |
| AmmoniumB | 24 h | 1035 | 10480 | 91 | 11 |
| UreaB | 24 h | 4115 | 14690 | 367 | 25923 |
| ControlB | 24 h | 3667 | 11867 | 311 | 26658 |
| AmmoniumC | 24 h | 3922 | 17544 | 347 | 34375 |
| NitrateC | 24 h | 4420 | 12412 | 263 | 25098 |
| ControlC | 24 h | 2685 | 12764 | 233 | 31 |
| UreaC | 24 h | 2299 | 8127 | 178 | 33 |

| **Enclosure** | **Interval** | **Candidatus Poribacteria** | **Chlamydiae** | **Chlorobi** | **Chloroflexi** |
| --- | --- | --- | --- | --- | --- |
| NitrateA | 0 h | 195 | 1132 | 2803 | 46 |
| ControlA | 0 h | 32 | 162 | 970 | 2903 |
| AmmoniumA | 0 h | 47 | 180 | 1098 | 3165 |
| UreaA | 0 h | 239 | 1408 | 3877 | 87 |
| NitrateB | 0 h | 223 | 1077 | 3031 | 64 |
| AmmoniumB | 0 h | 24 | 145 | 780 | 2371 |
| UreaB | 0 h | 121 | 808 | 2327 | 39 |
| ControlB | 0 h | 54 | 240 | 1139 | 3361 |
| AmmoniumC | 0 h | 187 | 985 | 3470 | 48 |
| NitrateC | 0 h | 118 | 734 | 2140 | 39 |
| ControlC | 0 h | 101 | 627 | 1515 | 38 |
| UreaC | 0 h | 87 | 445 | 1323 | 19 |
| NitrateA | 2 h | 26 | 116 | 614 | 1308 |
| ControlA | 2 h | 82 | 566 | 1456 | 28 |
| AmmoniumA | 2 h | 166 | 911 | 3114 | 61 |
| UreaA | 2 h | 96 | 717 | 2029 | 29 |
| NitrateB | 2 h | 32 | 159 | 779 | 2653 |
| AmmoniumB | 2 h | 45 | 171 | 1090 | 3746 |
| UreaB | 2 h | 148 | 590 | 3916 | 114 |
| ControlB | 2 h | 171 | 873 | 2490 | 49 |
| AmmoniumC | 2 h | 44 | 180 | 1255 | 3269 |
| NitrateC | 2 h | 80 | 561 | 1638 | 26 |
| ControlC | 2 h | 23 | 89 | 719 | 1812 |
| UreaC | 2 h | 42 | 171 | 865 | 2450 |
| NitrateA | 24 h | 27 | 65 | 429 | 1569 |
| ControlA | 24 h | 6 | 70 | 350 | 1000 |
| AmmoniumA | 24 h | 32 | 94 | 815 | 2725 |
| UreaA | 24 h | 72 | 529 | 1807 | 25 |
| NitrateB | 24 h | 121 | 1066 | 3362 | 70 |
| AmmoniumB | 24 h | 54 | 401 | 2033 | 28 |
| UreaB | 24 h | 79 | 229 | 1710 | 4821 |
| ControlB | 24 h | 48 | 212 | 1390 | 3588 |
| AmmoniumC | 24 h | 79 | 270 | 1938 | 6064 |
| NitrateC | 24 h | 70 | 228 | 1506 | 5056 |
| ControlC | 24 h | 146 | 843 | 3191 | 48 |
| UreaC | 24 h | 142 | 944 | 2583 | 35 |

| **Enclosure** | **Interval** | **C hysiogenetes** | **Cyanobacteria** | **Deferribacteres** | **Deinococcus-Thermus** |
| --- | --- | --- | --- | --- | --- |
| NitrateA | 0 h | 135 | 791220 | 86 | 872 |
| ControlA | 0 h | 50 | 741568 | 59 | 792 |
| AmmoniumA | 0 h | 47 | 732368 | 70 | 950 |
| UreaA | 0 h | 190 | 879241 | 139 | 1257 |
| NitrateB | 0 h | 221 | 843142 | 97 | 916 |
| AmmoniumB | 0 h | 39 | 548201 | 81 | 698 |
| UreaB | 0 h | 140 | 606872 | 60 | 724 |
| ControlB | 0 h | 46 | 656176 | 113 | 1048 |
| AmmoniumC | 0 h | 130 | 395704 | 119 | 1234 |
| NitrateC | 0 h | 119 | 603621 | 62 | 603 |
| ControlC | 0 h | 84 | 483371 | 65 | 466 |
| UreaC | 0 h | 69 | 426831 | 30 | 420 |
| NitrateA | 2 h | 29 | 506155 | 47 | 436 |
| ControlA | 2 h | 110 | 586549 | 36 | 496 |
| AmmoniumA | 2 h | 134 | 673389 | 78 | 863 |
| UreaA | 2 h | 140 | 656756 | 54 | 582 |
| NitrateB | 2 h | 49 | 607497 | 86 | 844 |
| AmmoniumB | 2 h | 65 | 798188 | 88 | 1096 |
| UreaB | 2 h | 3810 | 1443 | 1433 | 0 |
| ControlB | 2 h | 183 | 649598 | 74 | 842 |
| AmmoniumC | 2 h | 64 | 908756 | 88 | 997 |
| NitrateC | 2 h | 74 | 590763 | 34 | 500 |
| ControlC | 2 h | 32 | 660335 | 49 | 544 |
| UreaC | 2 h | 37 | 764965 | 55 | 735 |
| NitrateA | 24 h | 27 | 572339 | 43 | 423 |
| ControlA | 24 h | 27 | 291080 | 42 | 404 |
| AmmoniumA | 24 h | 45 | 934821 | 59 | 656 |
| UreaA | 24 h | 145 | 577123 | 50 | 605 |
| NitrateB | 24 h | 169 | 1062721 | 89 | 1103 |
| AmmoniumB | 24 h | 98 | 386476 | 32 | 631 |
| UreaB | 24 h | 76 | 1236329 | 138 | 1391 |
| ControlB | 24 h | 65 | 886108 | 116 | 1141 |
| AmmoniumC | 24 h | 116 | 1503347 | 123 | 1520 |
| NitrateC | 24 h | 84 | 1417963 | 95 | 1184 |
| ControlC | 24 h | 169 | 432294 | 78 | 1066 |
| UreaC | 24 h | 164 | 772430 | 62 | 752 |

| **Enclosure** | **Interval** | **Dictyoglomi** | **Elusimicrobia** | **Fibrobacteres** | **Firmicutes** |
| --- | --- | --- | --- | --- | --- |
| NitrateA | 0 h | 41 | 30 | 29 | 636715 |
| ControlA | 0 h | 45 | 23 | 20 | 124289 |
| AmmoniumA | 0 h | 67 | 59 | 28 | 20854 |
| UreaA | 0 h | 67 | 65 | 56 | 26252 |
| NitrateB | 0 h | 43 | 34 | 31 | 21940 |
| AmmoniumB | 0 h | 43 | 28 | 24 | 20384 |
| UreaB | 0 h | 39 | 27 | 21 | 17062 |
| ControlB | 0 h | 58 | 41 | 43 | 21210 |
| AmmoniumC | 0 h | 61 | 32 | 34 | 7706 |
| NitrateC | 0 h | 41 | 29 | 14 | 4136 |
| ControlC | 0 h | 27 | 26 | 30 | 14736 |
| UreaC | 0 h | 24 | 19 | 9 | 13624 |
| NitrateA | 2 h | 37 | 17 | 22 | 14963 |
| ControlA | 2 h | 22 | 17 | 15 | 16976 |
| AmmoniumA | 2 h | 47 | 23 | 28 | 16829 |
| UreaA | 2 h | 38 | 25 | 23 | 15778 |
| NitrateB | 2 h | 66 | 32 | 26 | 5750 |
| AmmoniumB | 2 h | 48 | 33 | 33 | 6510 |
| UreaB | 2 h | 18 | 0 | 110 | 467 |
| ControlB | 2 h | 54 | 24 | 31 | 22157 |
| AmmoniumC | 2 h | 54 | 39 | 32 | 25941 |
| NitrateC | 2 h | 30 | 8 | 17 | 19212 |
| ControlC | 2 h | 22 | 12 | 24 | 18772 |
| UreaC | 2 h | 44 | 23 | 28 | 20364 |
| NitrateA | 24 h | 14 | 8 | 15 | 4648 |
| ControlA | 24 h | 10 | 11 | 13 | 5500 |
| AmmoniumA | 24 h | 34 | 48 | 17 | 28922 |
| UreaA | 24 h | 18 | 25 | 22 | 36700 |
| NitrateB | 24 h | 51 | 37 | 27 | 31762 |
| AmmoniumB | 24 h | 19 | 17 | 18 | 56867 |
| UreaB | 24 h | 84 | 28 | 51 | 27964 |
| ControlB | 24 h | 59 | 41 | 40 | 26016 |
| AmmoniumC | 24 h | 85 | 38 | 34 | 11011 |
| NitrateC | 24 h | 79 | 41 | 38 | 9307 |
| ControlC | 24 h | 55 | 41 | 41 | 32024 |
| UreaC | 24 h | 23 | 34 | 23 | 33478 |

| **Enclosure** | **Interval** | **Fusobacteria** | **Gemmatimonadetes** | **Lentisphaerae** | **Nitrospirae** |
| --- | --- | --- | --- | --- | --- |
| NitrateA | 0 h | 160 | 1258 | 70 | 183 |
| ControlA | 0 h | 172 | 1353 | 71 | 181 |
| AmmoniumA | 0 h | 119 | 1741 | 81 | 224 |
| UreaA | 0 h | 242 | 2902 | 74 | 262 |
| NitrateB | 0 h | 87 | 1412 | 81 | 205 |
| AmmoniumB | 0 h | 90 | 1066 | 60 | 136 |
| UreaB | 0 h | 99 | 1211 | 70 | 171 |
| ControlB | 0 h | 156 | 2203 | 97 | 236 |
| AmmoniumC | 0 h | 131 | 2094 | 104 | 218 |
| NitrateC | 0 h | 79 | 1275 | 57 | 152 |
| ControlC | 0 h | 81 | 849 | 43 | 115 |
| UreaC | 0 h | 82 | 574 | 37 | 77 |
| NitrateA | 2 h | 89 | 1160 | 39 | 112 |
| ControlA | 2 h | 83 | 1049 | 42 | 114 |
| AmmoniumA | 2 h | 107 | 1934 | 77 | 154 |
| UreaA | 2 h | 77 | 1249 | 54 | 113 |
| NitrateB | 2 h | 107 | 1831 | 63 | 187 |
| AmmoniumB | 2 h | 148 | 2020 | 75 | 177 |
| UreaB | 2 h | 5612 | 385 | 29039 | 7 |
| ControlB | 2 h | 139 | 2507 | 67 | 175 |
| AmmoniumC | 2 h | 108 | 2388 | 52 | 162 |
| NitrateC | 2 h | 59 | 946 | 35 | 119 |
| ControlC | 2 h | 86 | 1305 | 39 | 142 |
| UreaC | 2 h | 102 | 1460 | 69 | 164 |
| NitrateA | 24 h | 88 | 874 | 33 | 90 |
| ControlA | 24 h | 111 | 744 | 25 | 99 |
| AmmoniumA | 24 h | 136 | 1731 | 52 | 174 |
| UreaA | 24 h | 82 | 1019 | 32 | 121 |
| NitrateB | 24 h | 115 | 2190 | 65 | 183 |
| AmmoniumB | 24 h | 170 | 827 | 31 | 77 |
| UreaB | 24 h | 199 | 3490 | 91 | 315 |
| ControlB | 24 h | 130 | 2659 | 102 | 292 |
| AmmoniumC | 24 h | 140 | 3858 | 111 | 335 |
| NitrateC | 24 h | 142 | 2705 | 94 | 266 |
| ControlC | 24 h | 120 | 2527 | 87 | 212 |
| UreaC | 24 h | 116 | 1324 | 52 | 138 |

| **Enclosure** | **Interval** | **Planctomycetes** | **Proteobacteria** | **Spirochaetes** | **Synergistetes** |
| --- | --- | --- | --- | --- | --- |
| NitrateA | 0 h | 5747 | 81826 | 381 | 63 |
| ControlA | 0 h | 4315 | 69173 | 375 | 83 |
| AmmoniumA | 0 h | 7547 | 88478 | 472 | 107 |
| UreaA | 0 h | 10267 | 103050 | 553 | 135 |
| NitrateB | 0 h | 5793 | 89606 | 419 | 99 |
| AmmoniumB | 0 h | 6047 | 62231 | 311 | 76 |
| UreaB | 0 h | 6801 | 65069 | 372 | 76 |
| ControlB | 0 h | 12610 | 85727 | 495 | 120 |
| AmmoniumC | 0 h | 15839 | 89294 | 543 | 127 |
| NitrateC | 0 h | 3837 | 62152 | 314 | 65 |
| ControlC | 0 h | 4048 | 45913 | 281 | 71 |
| UreaC | 0 h | 5204 | 27820 | 216 | 39 |
| NitrateA | 2 h | 3792 | 45781 | 252 | 48 |
| ControlA | 2 h | 3375 | 42974 | 248 | 46 |
| AmmoniumA | 2 h | 7818 | 83133 | 351 | 95 |
| UreaA | 2 h | 4228 | 60353 | 287 | 65 |
| NitrateB | 2 h | 8608 | 73339 | 336 | 76 |
| AmmoniumB | 2 h | 10991 | 92483 | 444 | 88 |
| UreaB | 2 h | 15 | 8230 | 153 | 73 |
| ControlB | 2 h | 5507 | 80032 | 434 | 85 |
| AmmoniumC | 2 h | 7070 | 99677 | 538 | 93 |
| NitrateC | 2 h | 2971 | 49797 | 252 | 64 |
| ControlC | 2 h | 3379 | 57130 | 267 | 47 |
| UreaC | 2 h | 6510 | 62002 | 348 | 81 |
| NitrateA | 24 h | 2935 | 63610 | 202 | 51 |
| ControlA | 24 h | 1705 | 52863 | 154 | 32 |
| AmmoniumA | 24 h | 4543 | 90107 | 350 | 81 |
| UreaA | 24 h | 3256 | 85157 | 195 | 52 |
| NitrateB | 24 h | 6537 | 118219 | 443 | 97 |
| AmmoniumB | 24 h | 3969 | 81774 | 152 | 56 |
| UreaB | 24 h | 8707 | 152063 | 688 | 139 |
| ControlB | 24 h | 7216 | 121314 | 505 | 119 |
| AmmoniumC | 24 h | 6544 | 192078 | 691 | 139 |
| NitrateC | 24 h | 6683 | 134278 | 624 | 105 |
| ControlC | 24 h | 9090 | 114101 | 406 | 104 |
| UreaC | 24 h | 4726 | 78531 | 308 | 66 |

| **Enclosure** | **Interval** | **Tenericutes** | **Thermotogae** | **Verrucomicrobia** | **unclassified (derived from Bacteria)** |
| --- | --- | --- | --- | --- | --- |
| NitrateA | 0 h | 34 | 211 | 1903 | 425 |
| ControlA | 0 h | 42 | 204 | 1880 | 185 |
| AmmoniumA | 0 h | 56 | 185 | 2548 | 177 |
| UreaA | 0 h | 76 | 275 | 3143 | 208 |
| NitrateB | 0 h | 49 | 201 | 2192 | 173 |
| AmmoniumB | 0 h | 61 | 178 | 1969 | 148 |
| UreaB | 0 h | 39 | 166 | 1888 | 127 |
| ControlB | 0 h | 43 | 256 | 3034 | 222 |
| AmmoniumC | 0 h | 44 | 272 | 2867 | 223 |
| NitrateC | 0 h | 47 | 175 | 1451 | 102 |
| ControlC | 0 h | 18 | 115 | 1211 | 89 |
| UreaC | 0 h | 33 | 98 | 1068 | 72 |
| NitrateA | 2 h | 19 | 117 | 1211 | 71 |
| ControlA | 2 h | 31 | 125 | 1101 | 82 |
| AmmoniumA | 2 h | 69 | 169 | 1874 | 157 |
| UreaA | 2 h | 26 | 162 | 1160 | 108 |
| NitrateB | 2 h | 46 | 181 | 1959 | 135 |
| AmmoniumB | 2 h | 41 | 274 | 2356 | 200 |
| UreaB | 2 h | 84575 | 46 | 1023 | 63 |
| ControlB | 2 h | 42 | 178 | 1864 | 136 |
| AmmoniumC | 2 h | 46 | 219 | 1833 | 131 |
| NitrateC | 2 h | 19 | 121 | 1002 | 96 |
| ControlC | 2 h | 35 | 122 | 1162 | 94 |
| UreaC | 2 h | 29 | 171 | 1556 | 117 |
| NitrateA | 24 h | 21 | 100 | 839 | 120 |
| ControlA | 24 h | 22 | 45 | 596 | 436 |
| AmmoniumA | 24 h | 50 | 210 | 1104 | 171 |
| UreaA | 24 h | 27 | 117 | 897 | 154 |
| NitrateB | 24 h | 45 | 234 | 2008 | 203 |
| AmmoniumB | 24 h | 15 | 127 | 845 | 2979 |
| UreaB | 24 h | 67 | 324 | 2726 | 246 |
| ControlB | 24 h | 59 | 276 | 2556 | 233 |
| AmmoniumC | 24 h | 75 | 331 | 2265 | 268 |
| NitrateC | 24 h | 52 | 232 | 2676 | 207 |
| ControlC | 24 h | 35 | 214 | 2347 | 206 |
| UreaC | 24 h | 30 | 154 | 1540 | 169 |

| **Enclosure** | **Interval** | **Sum** |
| --- | --- | --- |
| NitrateA | 0 h | 1538644 |
| ControlA | 0 h | 980079 |
| AmmoniumA | 0 h | 896645 |
| UreaA | 0 h | 1053895 |
| NitrateB | 0 h | 985002 |
| AmmoniumB | 0 h | 668673 |
| UreaB | 0 h | 716999 |
| ControlB | 0 h | 822603 |
| AmmoniumC | 0 h | 546243 |
| NitrateC | 0 h | 690976 |
| ControlC | 0 h | 561922 |
| UreaC | 0 h | 484948 |
| NitrateA | 2 h | 593411 |
| ControlA | 2 h | 662337 |
| AmmoniumA | 2 h | 808327 |
| UreaA | 2 h | 752877 |
| NitrateB | 2 h | 729402 |
| AmmoniumB | 2 h | 951679 |
| UreaB | 2 h | 164094 |
| ControlB | 2 h | 779513 |
| AmmoniumC | 2 h | 1089439 |
| NitrateC | 2 h | 675566 |
| ControlC | 2 h | 763710 |
| UreaC | 2 h | 884537 |
| NitrateA | 24 h | 662295 |
| ControlA | 24 h | 374604 |
| AmmoniumA | 24 h | 1090947 |
| UreaA | 24 h | 718886 |
| NitrateB | 24 h | 1247743 |
| AmmoniumB | 24 h | 549313 |
| UreaB | 24 h | 1487050 |
| ControlB | 24 h | 1096788 |
| AmmoniumC | 24 h | 1787648 |
| NitrateC | 24 h | 1625908 |
| ControlC | 24 h | 615158 |
| UreaC | 24 h | 908461 |

**Figure S1.** Scatter plots for the nutrient treatments comparing the 0 h gene expression values to the 2 h gene expression values. Green dots are genes that were significantly upregulated, red dots are genes that were significantly downregulated, and gray dots are genes that had no significant change in gene expression.

**Figure S2.** Scatter plots for the nutrient treatments comparing the 0 h gene expression values to the 24 h gene expression values. Green dots are genes that were significantly upregulated, red dots are genes that were significantly downregulated, and gray dots are genes that had no significant change in gene expression.

**Figure S3.** Heatmap for the expression (log[x + 1]) of nitrogen acquisition and metabolism genes of *Microcystis* sp. Dendrogram displaying the clustering of individual samples (*n* = 36) was determined using a Bray-Curtis similarity analysis. Each sample is unique to a specific enclosure and sampling timepoint (Treatment, timepoint sampled, replicate).

**Figure S4.** Heatmap for the expression (log[x + 1]) of pigment associated genes of *Microcystis* sp. Dendrogram displaying the clustering of individual samples (*n =* 36) was determined using a Bray-Curtis similarity analysis. Each sample is unique to a specific enclosure and sampling timepoint (Treatment, timepoint sampled, replicate).
